# Scoring for Life: The Legacy of Virginia Apgar in Neonatal Medicine

**DOI:** 10.7759/cureus.69281

**Published:** 2024-09-12

**Authors:** Ahmed Elashmawy, Anas Haq, Angelo Federico, Mohammadali Chokr, Ali J Bazzi

**Affiliations:** 1 Pediatrics, Wayne State University School of Medicine, Detroit, USA; 2 Pediatrics, Children's Hospital of Michigan, Detroit, USA; 3 General Surgery, Corewell Health, Dearborn, USA; 4 Anesthesia, Corewell Health East, Dearborn, USA; 5 Pediatrics, Corewell Health East, Dearborn, USA

**Keywords:** biographies, historical vignettes, medical innovation, medical stories, neonatal medicine

## Abstract

Dr. Virginia Apgar was a pioneering figure in obstetric anesthesiology and neonatology, breaking significant barriers in the medical field. She developed a standardized method for assessing the health of newborns, which has become a global standard and continues to be widely used even today. In addition to her clinical innovations, Dr. Apgar was a dedicated educator and advocate for prenatal care and the prevention of birth defects. Her contributions to medicine have had a lasting impact on healthcare practices worldwide.

Beyond her clinical innovations, Dr. Apgar was a trailblazer in the medical field, breaking through gender barriers at a time when opportunities for women in medicine were severely limited. She also later became a key advocate for public health initiatives focused on reducing birth defects and improving maternal and infant care. Dr. Apgar's career was marked by her dedication to advancing medical knowledge, her commitment to education, and her tireless efforts to improve healthcare practices. Her legacy continues to inspire healthcare professionals around the world, underscoring the enduring value of her contributions to medicine.

## Introduction and background

Dr. Virginia Apgar (MD) was a monumental pioneer in the fields of anesthesiology and neonatology. She developed the Apgar score, the first standardized method for assessing a newborn's health immediately after birth. Born in Westfield, New Jersey, on June 7, 1909, Apgar was inspired by her father's scientific interests and the health challenges faced by her siblings. These early experiences motivated her to pursue a career in medicine [1-3].

Apgar attended Mount Holyoke College, graduating in 1929 with a degree in zoology. She then enrolled at the College of Physicians and Surgeons at Columbia University, where she excelled in her studies and graduated fourth in her class in 1933. Initially aspiring to become a surgeon, she faced numerous gender-based barriers and was encouraged by a mentor, Dr. Alan Whipple, to consider anesthesiology instead [1-3].

In 1938, Apgar became the first woman to head the division of anesthesiology at Columbia University. By 1949, she had become the institution’s first female professor. Her research focused on the effects of anesthesia on newborns, leading to the creation of the Apgar score in 1952. This five-point scoring system evaluates newborns based on skin color, pulse, reflex, muscle tone, and respirations and is still used by physicians to assess a newborn’s immediate need for medical intervention (Table 1) [1,2].

**Table 1:** A detailed breakdown of the Apgar scoring system, with criteria, features, and scoring

Criterion	Feature/modality	Score of 0	Score of 1	Score of 2
Appearance (skin color)	Skin color and oxygenation	Blue/pale all over	Pink body, blue extremities	Pink all over
Pulse (heart rate)	Heart rate (beats per minute)	Absent (0)	Below 100	Above 100
Grimace (reflex irritability)	Response to stimulation	No response	Grimace/weak response	Cough, sneeze, or vigorous cry
Activity (muscle tone)	Muscle tone	Limp	Some flexion of extremities	Active motion (well-flexed)
Respiration (breathing effort)	Breathing effort and rate	Absent	Slow/irregular breathing	Good, strong cry

## Review

Early career and challenges

Virginia Apgar embarked on her medical journey at a time when the field of medicine was overwhelmingly male-dominated. After earning her undergraduate degree in zoology from Mount Holyoke College, she entered the College of Physicians and Surgeons at Columbia University in 1929. Her dedication and academic excellence led her to graduate fourth in her class in 1933, which was a remarkable achievement for any student, especially a woman during that era [1,2].

Despite her academic success, Apgar faced significant barriers when she aspired to become a surgeon. At the time, surgery was a notoriously difficult field for women to enter and advance within, due to societal expectations and professional biases that severely limited opportunities in surgical specialties. Recognizing both her potential and the era's limitations, her mentor, Dr. Alan Whipple, advised her to pursue anesthesiology, a field that was less established but rich with potential for growth and innovation [4,5]. This advice proved pivotal. Anesthesiology, still emerging as a distinct medical specialty, offered Apgar the opportunity to pioneer new approaches and techniques. By 1938, she became the first woman to head the division of anesthesiology at Columbia University. Her early work focused on the effects of anesthesia during childbirth, a critically important but under-researched area at the time. Through her innovative approach, she brought much-needed rigor to the field, aiming to improve the safety and outcomes in relation to both mothers and newborns during labor [5,6].

Contributions to anesthesiology and neonatology

Virginia Apgar’s most enduring contribution to medicine is the Apgar score, introduced in 1952. Before this, there was no standardized method for assessing the health of newborns immediately after birth, leading to inconsistent care and delayed interventions for infants in distress. Apgar identified the need for a quick, reliable, and objective evaluation method in the crucial minutes following delivery [1,5].

**Figure 1:**
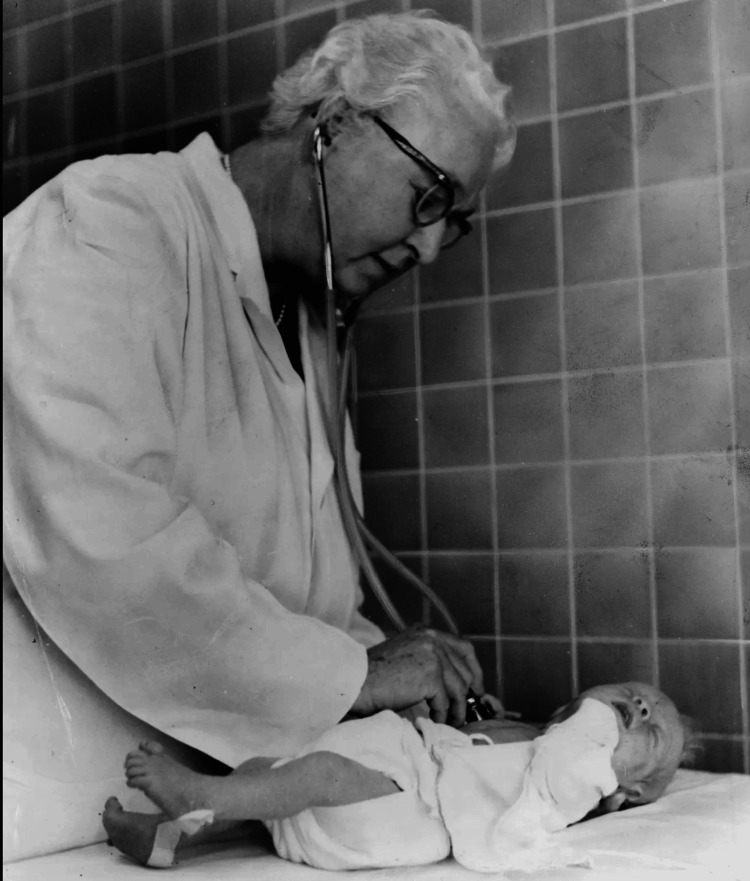
Virginia Apgar examining a baby, 1966 This photograph, taken by Al Ravenna for the World Journal Tribune, is in the public domain. It is available through the Library of Congress. As a public domain image, no further permissions are required for its use [11].

The Apgar score assesses five key criteria: appearance (skin color), pulse (heart rate), grimace response (reflexes), activity (muscle tone), and respiration (breathing effort). Each criterion is scored on a scale of 0 to 2, with a maximum total score of 10 [7]. This system enables healthcare providers to rapidly determine whether a newborn requires immediate medical attention, such as resuscitation. The simplicity and effectiveness of the Apgar score led to its swift adoption worldwide, becoming a standard part of neonatal care [6,8]. The introduction of the Apgar Score had a profound impact on neonatal medicine. By providing a consistent assessment method to assess newborns (Figure 1), it significantly enhanced healthcare providers' ability to identify infants at risk and intervene promptly, thereby reducing neonatal mortality and morbidity. While research shows that low Apgar scores at five minutes are associated with higher risks of neonatal mortality and long-term neurological issues like cerebral palsy, the majority of infants with low scores develop normally. This underscores the Apgar score's value as an initial assessment tool rather than a definitive predictor of outcomes [9,10].

Beyond the Apgar score, Apgar’s work was instrumental in establishing anesthesiology as a respected medical specialty. She strongly advocated for specialized training in anesthesiology, emphasizing the importance of expertise in ensuring patient safety and improving outcomes, particularly in obstetric anesthesia. Her efforts played a key role in the formal recognition of anesthesiology as a critical component of surgical and obstetric care, laying the foundation for modern practices [4,5].

Advocacy for public health and later career

In 1959, after nearly two decades of groundbreaking work in anesthesiology and neonatology, Apgar shifted her focus to public health. Motivated by a desire to address broader issues in maternal and infant health, she earned a master’s degree in public health from Johns Hopkins University. She then joined the National Foundation for Infantile Paralysis, now known as the March of Dimes, where she became a leading advocate for preventing birth defects and improving prenatal care [2,8]. At the March of Dimes, Apgar spearheaded numerous public health initiatives aimed at reducing the incidence of birth defects. She was particularly passionate about educating the public on the importance of early prenatal care and the role of environmental and genetic factors in fetal development. Her advocacy was instrumental in raising awareness about birth defects and promoting research into their causes and prevention [8].

Throughout her career, Apgar received numerous accolades. She was the first woman to serve on the Executive Committee of the American Society of Anesthetists, acting as Treasurer, and in 1949, she became the first woman appointed as a professor of anesthesiology at Columbia University College of Physicians and Surgeons. Apgar later earned her master's degree in public health and joined the faculty at Johns Hopkins University School of Public Health as a professor in the Department of Genetics. In 1961, she became the first woman to receive the Distinguished Service Award, the highest honor of the American Society of Anesthetists [12].

Apgar’s impact on public health extended beyond the initiatives she led. She was a powerful advocate for improving maternal and infant health, supporting policies and programs that promoted research, education, and healthcare services for mothers and children. Dr. Virginia Apgar's work laid the foundation for several key public health practices still in use today, including the development of neonatal intensive care units (NICUs), the implementation of newborn screening programs, and the establishment of standardized perinatal care guidelines [2]. Her advocacy also influenced public health campaigns promoting prenatal care and contributed to maternal and child health programs that emphasize early intervention and comprehensive care. These initiatives reflect her enduring impact on improving maternal and infant health outcomes. After decades of honors and awards, in 1973, she coauthored the book,* Is My Baby All Right?* She died shortly after in 1974 [13].

In addition to her professional achievements, Apgar was a trailblazer for women in medicine. At a time when only a few women held leadership positions in the medical field, she demonstrated that women could excel and lead in even the most challenging and technical areas of medicine. Her success paved the way for future generations of women in medicine, challenging and changing societal perceptions about women’s capabilities in professional and academic spheres.

## Conclusions

There is no denying the massive impact Dr. Virginia Apgar has had on the field of medicine. Her development of the Apgar score reshaped neonatal care and continues to be an incredibly useful tool that is utilized daily to assess the health of newborns as they enter the world. Beyond her work in neonatology, her contributions not only advanced medical practice but also challenged and changed societal perceptions about the capabilities of women in professional spheres and academia. Dr. Apgar's work is a demonstration of pure innovation and dedication. The enduring value of her work in improving maternal and child health and the impact she had continue to inspire and guide new generations of healthcare professionals to this day.
